# Gender Differences in S-Nitrosoglutathione Reductase Activity in the Lung

**DOI:** 10.1371/journal.pone.0014007

**Published:** 2010-11-16

**Authors:** Kathleen Brown-Steinke, Kimberly deRonde, Sean Yemen, Lisa A. Palmer

**Affiliations:** Department of Pediatrics, University of Virginia Health System, Charlottesville, Virginia, United States of America; University of Alabama-Birmingham, United States of America

## Abstract

S-nitrosothiols have been implicated in the etiology of various pulmonary diseases. Many of these diseases display gender preferences in presentation or altered severity that occurs with puberty, the mechanism by which is unknown. Estrogen has been shown to influence the expression and activity of endothelial nitric oxide synthase (eNOS) which is associated with increased S-nitrosothiol production. The effects of gender hormones on the expression and activity of the de-nitrosylating enzyme S-nitrosoglutathione reductase (GSNO-R) are undefined. This report evaluates the effects of gender hormones on the activity and expression of GSNO-R and its relationship to N-acetyl cysteine (NAC)-induced pulmonary hypertension (PH). GSNO-R activity was elevated in lung homogenates from female compared to male mice. Increased activity was not due to changes in GSNO-R expression, but correlated with GSNO-R S-nitrosylation: females were greater than males. The ability of GSNO-R to be activated by S-nitrosylation was confirmed by: 1) the ability of S-nitrosoglutathione (GSNO) to increase the activity of GSNO-R in murine pulmonary endothelial cells and 2) reduced activity of GSNO-R in lung homogenates from eNOS^−/−^ mice. Gender differences in GSNO-R activity appear to explain the difference in the ability of NAC to induce PH: female and castrated male animals are protected from NAC-induced PH. Castration results in elevated GSNO-R activity that is similar to that seen in female animals. The data suggest that GSNO-R activity is modulated by both estrogens and androgens in conjunction with hormonal regulation of eNOS to maintain S-nitrosothiol homeostasis. Moreover, disruption of this eNOS-GSNO-R axis contributes to the development of PH.

## Introduction

S-nitrosylation, a redox-based modification of a cysteine thiol by nitric oxide, is a post translational modification that can alter a protein's function. Mechanisms that control the addition and/or removal of the NO group from cysteine thiols are essential in determining the net effect of this modification. Formation of endogenous S-nitrosothiols can be mediated through: 1) the activity of any one of the nitric oxide synthase (NOS) isoforms, 2) oxidative reactions generating nitrosative species (for example, Fe^+3^NO, N_2_O_3_,) or 3) transnitrosative reactions (NO^+^-transfer) [1]–[3]. The production of S-nitrosothiols is opposed by mechanisms mediating de-nitrosylation which can: 1) occur non-enzymatically via homolytic or heterolytic cleavage, 2) be catalyzed by transition metal ions and reactive oxygen species, or 3) occur through enzymatic degradation [1], [4]. One specific enzyme that regulates S-nitrosothiol catabolism is S-nitrosoglutathione reductase (GSNO-R), a ubiquitously expressed NADH-dependent enzyme [2]. GSNO-R is responsible for the breakdown of S-nitrosoglutathione (GSNO) to oxidized glutathione and ammonia [5], [6]. Although the primary substrate for GSNO-R is GSNO [5], [6], the levels of other S-nitrosylated-proteins are affected indirectly through altered transnitrosation equilibria with GSNO.

S-nitrosothiols have been implicated in pulmonary diseases such as cystic fibrosis [3], [7]–[9], pulmonary hypertension [3], [7], [10], [11] and asthma [3], [7], [12], [13]. All of these pulmonary diseases display distinct gender preferences in presentation or a change in disease severity that occurs at puberty, the cause of which is unknown [14]–[17]. Gender differences in the activity and/or expression of GSNO-R have been suggested. Gastric activity of GSNO-R may be a component of the enhanced vulnerability of women to develop alcohol–related diseases [18]. Likewise, gender differences seen in the lipopolysaccharide (LPS) model of septic shock are eliminated in GSNO-R knockout mice [6]. To date, the influence of de-nitrosylation on the gender predilection of these lung diseases has not been addressed. The current studies evaluate the relationship between gender and the activity and/or expression of GSNO-R in the lung. The data demonstrate that GSNO-R activity is elevated in the female mouse lung when compared to the male. This increased activity does not reflect differences in GSNO-R protein expression, but rather, reflects differences both in endothelial nitric oxide synthase (eNOS)-dependent GSNO-R S-nitrosylation and androgen exposure. Indeed, mice deficient in eNOS have reduced GSNO-R activity, and S-nitrosylation increases GSNO-R activity, suggesting that estrogen-dependent increases in eNOS lead to increased GSNO-R activity in the female lung, protecting against excessive S-nitrosylation. Lastly, this gender discordance in the eNOS/GSNO-R axis is relevant to pulmonary biology. Female mice are protected from the physiological effects mediated by the conversion of N-acetyl cysteine (NAC) to S-nitroso-N-acetyl cysteine (SNOAC) in vivo, despite increased eNOS expression. However, they develop hypoxia-mimetic pulmonary hypertension (PH) in response to chronic SNOAC exposure like their male littermates. This observation may have implications for human disease. For example, increased eNOS expression in females could predispose them to PH if the counter-regulatory GSNO-R response is abnormal.

## Results

### GSNO-R activity, not protein expression, is greater in lung homogenates of female than male mice

**Figure 1:**
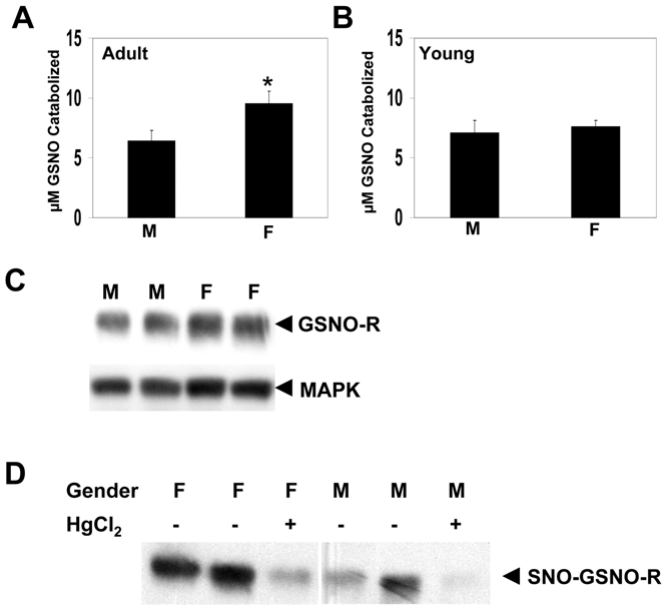
GSNO-R activity and S-nitrosylation is elevated in the female mouse lung. (A) GSNO-R activity was measured in lung homogenates from male and female C57Bl6/129SvEv mice using LC/MS. GSNO-R activity is expressed as the amount of GSNO catabolized after 5 min. Activity present in female mouse lung homogenates was approximately two times that seen in the males (n = 20, p<0.003). (B) GSNO-R activity was measured by LC/MS in lung homogenates from young (4 w) C57Bl6/129SvEv sexually immature male and female mice. Gender differences in GSNO-R activity were not seen in the young animals (n = 5). (C) GSNO-R protein expression was determined in adult C57Bl6/129SvEv male and female lung homogenates. Protein expression was not significantly different between genders. (D) Lung homogenates from C57Bl6/129SvEv male and female mice were subjected to biotin switch to determine if GSNO-R was S-nitrosylated in vivo. Lung homogenates from both male and female animals demonstrate the presence of GSNO-R S-nitrosylation. However, the extent of S-nitrosylation was greater in the female animals. Incubation of the lung homogenates with mercuric chloride reduced the abundance of S-nitrosylated GSNO-R.

Initial studies evaluated the activity and expression of GSNO-R in lung homogenates in male and female mice. GSNO-R activity was evaluated using liquid chromatography/mass spectroscopy (LC/MS). GSNO-R activity measured by LC/MS was significantly higher in adult (10–12 w) female animals compared to their corresponding adult male counterparts (Figure 1A). Similar gender specific differences (2–3 fold) were detected when GSNO-R activity was measured by GSNO-dependent NADH consumption or modified Saville Assay (Tables 1,2,3) In contrast, no significant differences in GSNO-R activity were seen in the lungs of young (4 w) male and female animals (Figure 1B). To determine if this gender difference was specific for the lung, GSNO-R activity was measured in the liver and the kidney. Unlike the lung, no gender specific differences in GSNO-R activity were seen in either tissue (Tables 1 and 2). To determine if the differences in GSNO-R activity in the adult murine lung were due to changes in protein expression, Western blot analysis was performed (Figure 1C). No significant differences in GSNO-R protein expression were seen in the lung homogenates of adult male and female animals.

**Table 1:** GSNO-R Activity determined by GSNO-dependent NADH Consumption is greater in lung homogenates obtained from female mice.

	GSNO-dependent NADH Consumption (µM NADH/min/mg protein)		
	Male	Female	Female: Male Ratio
Lung	5.51+/−2.9	10.58+/−3.6	1.92
Liver	28.1+/−3.77	19.46+/−5.75	0.70
Kidney	39.96+/−8.68	34.32+/−9.11	0.85

GSNO-R activity was measured in lung, liver, and kidney homogenates obtained from adult (10–12 w) male and female mice using a GSNO-dependent NADH Consumption assay. NADH consumption was evaluated in homogenates (0.3 µg) in the absence or presence of 100 µM GSNO. Gender specific differences were seen in the lung homogenates (n = 4). No gender specific differences were seen with either the liver or the kidney homogenates (n = 2–4).

**Table 2:** GSNO-R activity measured by modified Saville Assay is greater in lung homogenates obtained from female mice.

	NADH-Dependent GSNO-R Activity (µM GSNO/min/mg protein)		
	Male	Female	Female: Male Ratio
Lung	2.56+/−0.20	5.32+/−1.06	2.07
Liver	6.24+/−1.96	6.14+/−0.25	0.98
Kidney	5.68+/−0.69	6.06+/−1.1	1.10

GSNO-R activity was determined in homogenates from the lung, liver and kidney of adult male and female mice. GSNO-R activity was measured in homogenates (250 µg) in the presence of 100 µM GSNO in the presence or absence of NADH at 37°C for 5 min. GSNO-R activity was significantly higher in lung homogenates obtained from female mice compared to male mice (n = 4). No gender specific differences were seen in either the liver or kidney homogenates (n = 2–4).

**Table 3:** Comparison of Methods used to Measure NADH-Dependent GSNO-R Activity.

Gender	[GSNO] µM	GSNO-R activityµM/min/mg protein	Protein precipitation	Female:Male ratio
Female	28	5.01+/−1.51	N	
Male	28	2.37+/−0.35	N	2.11
Female	28	3.94+/−1.52	Y	
Male	28	1.16+/−0.33	Y	3.40
Female	100	8.41+/−2.69	N	
Male	100	3.06+/−0.52	N	2.75
Female	100	5.32+/−1.06	Y	
Male	100	2.56+/−0.20	Y	2.07

Lungs were harvested from adult (10–12 w) C57Bl6/129SeEv mice. Lung homogenates were subjected to NADH-dependent GSNO-R activity. The assay was performed as described in Methods. The effect of altering the concentration of GSNO in the assay and the precipitation of protein prior to measurement were compared in the same samples (n = 4). Note that higher activity was obtained in the absence of protein precipitation for both GSNO concentrations. Highest GSNO-R activity was obtained in the presence of 100 µM GSNO. GSNO-R activity was found to be 2–3 fold higher in the lung homogenates from female animals regardless of the method used to measure activity.

### GSNO-R S-nitrosylation is greater in female than male mice

The detected difference in GSNO-R activity present in the lung homogenates of male and female animals could be explained by differences in S-nitrosylation. To examine if GSNO-R is an S-nitrosylated protein, lung homogenates obtained from male and female animals were subjected to biotin switch followed by Western blot analysis (Figure 1D). GSNO-R was found to be S-nitrosylated in both male and female lung homogenates. The level of S-nitrosylation was greater in lung homogenates of female mice as compared to lung homogenates from male animals. S-nitrosylation was confirmed by the ability of mercuric chloride to significantly reduce the appearance of S-nitrosylated GSNO-R.

### S-nitrosoglutathione activates GSNO-R

**Figure 2:**
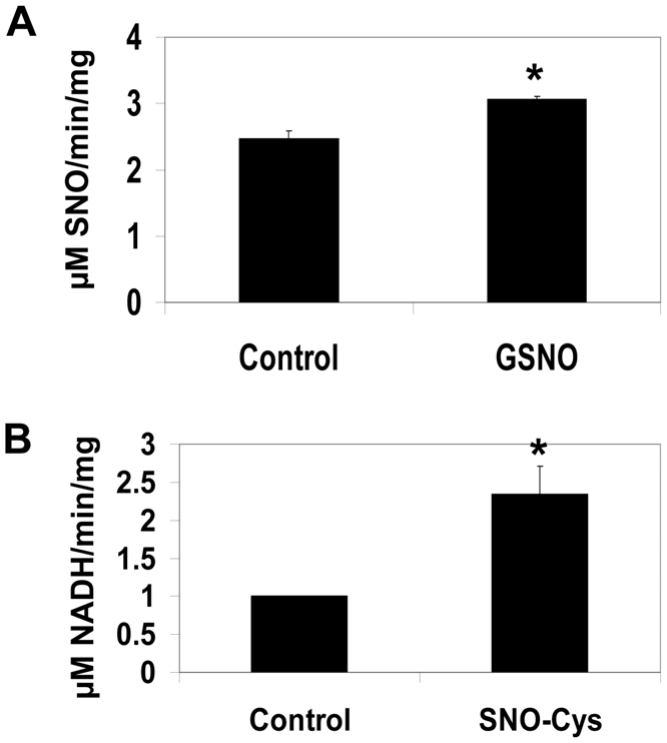
S-nitrosoglutatione activates GSNO-R. (A) Primary mouse lung endothelial cells were treated with 10 µM GSNO for 5 min and the activity of GSNO-R determined using a modified Saville Assay using 28 µM GSNO. GSNO-R activity was significantly increased after treatment of GSNO. (n = 3, p<0.011). (B) Cell lysates obtained from mouse lung endothelial cells were treated with or without 5 µM L-SNO-cysteine for 5 min. GSNO-R activity was measured by L-SNO-cysteine dependent NADH consumption. L-SNO-Cysteine resulted in a 2 fold increase in NADH consumption (n = 4, p<0.011).

Post-translational modifications by S-nitrosylation often results in a change in a protein's activity. Previous data demonstrate lung homogenates obtained from female animals have greater GSNO-R activity and elevated GSNO-R S-nitrosylation compared to male animals, suggesting that S-nitrosylation and activity are related. To define the impact of S-nitrosylation on the activity of GSNO-R, murine pulmonary endothelial cells were treated with and without 10 µM GSNO for 5 min. GSNO-R activity was measured in cell homogenates obtained from the untreated and treated cells. Treatment of murine lung endothelial cells with GSNO resulted in a significant increase in GSNO-R activity (Figure 2A). To determine if GSNO-R can be directly S-nitrosylated, cell lysates from murine pulmonary endothelial cells were treated with or without 5 µM L-SNO-cysteine (L-SNO-Cys) for 5 minutes and GSNO-R activity measured using alterations in NADH consumption. L-SNO-Cys was found to significantly increase NADH consumption approximately 2 fold (Figure 2B). This increase in NADH consumption with L-SNO-Cys was independent of gender.

### Endothelial nitric oxide synthase increases GSNO-R Activity

**Figure 3:**
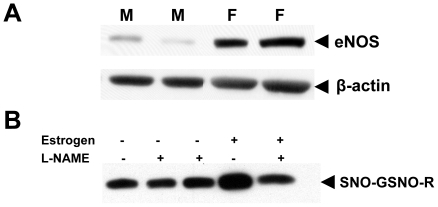
Estrogen Activation of GSNO-R is eNOS dependent. (A) Abundance of eNOS present in lung homogenates from male and female C57Bl6/129SvEv mice was determined by Western blot analysis using antibodies directed against eNOS and β-actin. eNOS protein levels were greater in lung homogenates of female animals. (B) Mouse lung endothelial cells isolated from female mouse lungs were treated with or without 10 µM estrogen in the presence or absence of 100 µM L-NAME for 4 h. Estrogen resulted in an increase in GSNO-R S-nitrosylation. The increased in GSNO-R S-nitrosylation was abrogated by pretreatment with L-NAME.

**Figure 4:**
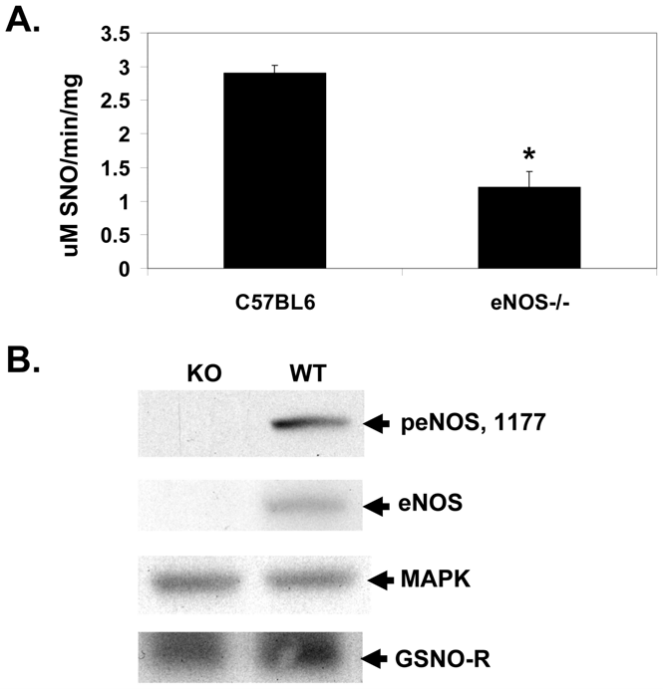
eNOS is required for GSNO-R activity. (A) GSNO-R activity was measured in the lung homogenates obtained from eNOS^−/−^ and wild type (C57Bl6) mice using a modified Saville Assay using 28 µM GSNO with no protein precipitation. GSNO-R activity was reduced by 50% in the eNOS^−/−^ lung homogenate compared to that seen in the wild type mice (n = 5–6, p<0.002). (B) Western blot analysis of GSNO-R protein present in wild type (C57Bl6) and eNOS^−/−^ mouse lungs. No significant differences were detected in GSNO-R protein levels (n = 3).

eNOS is the prominent isoform present in the pulmonary vascular endothelium. Estrogen increases the expression and activity of eNOS [19], [20]. Consistent with this observation, lung homogenates from female mice contained greater eNOS protein expression when compared to lung homogenates from male mice (Figure 3A). Increases in NOS activity are associated with increased formation of S-nitrosothiols. To determine if the increase in GSNO-R activity in the lungs of female animals is due to estrogen-induced increases in eNOS activity, murine pulmonary endothelial cells isolated from female mice were treated with 10 nM estrogen in the absence or presence of L-NAME. Estrogen increased the level of S-nitrosylated GSNO-R compared to control cells (Figure 3B). This increase was abrogated in the presence of L-NAME. To further confirm the effects of eNOS on the activity of GSNO-R, GSNO-R activity was examined in lung homogenates of eNOS deficient (eNOS^−/−^) mice using the modified Saville Assay (Figure 4A). Consistent with a role for eNOS in regulating the activity of GSNO-R, GSNO-R activity was found to be reduced by approximately 50% in eNOS^−/−^ animals compared to wild type control animals. The reduction in GSNO-R activity was not mediated by a decrease in GSNO-R expression (Figure 4B).

### Female mice are protected from increases in right ventricular weight and right ventricular pressure with NAC

**Figure 5:**
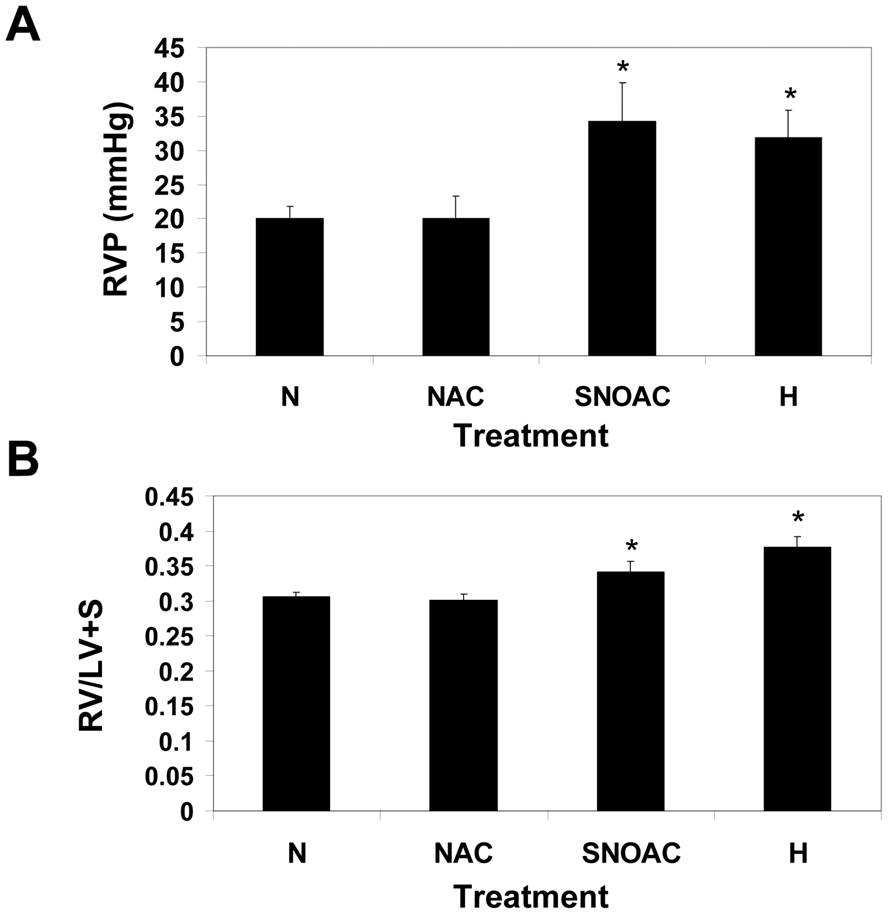
Female C57BL6/129SvEv mice do not develop PH with chronic, systemic administration of N-acetyl cysteine. C57Bl/129SvEv mice were untreated (N) or treated with 10 mg/ml NAC (NAC), 52 mM SNOAC (SNOAC) or hypoxia (H) for a period of 3 weeks. (A) Right ventricular pressure (RVP) and (B) right heart weight (expressed as right ventricular weight/left ventricular weight + septum weight (RV/LV+S) were determined. Female mice responded to only to SNOAC and hypoxia. * (*n = 19–25*, p<0.05).

NAC was found in increase right ventricular pressure and right ventricular weight in C57BL6/129SEV male mice in a manner that was indistinguishable from that induced by hypoxia [11]. Published data indicate that female gender is less susceptible to hypoxia induced PH (21–23). To determine if the female gender is less susceptible to NAC-induced PH, C57BL6/129SvEv mice were subjected to normoxia, 10 mg/ml NAC, 1 mg/ml SNOAC, or hypoxia for a period of three weeks [11]. In contrast to the male animals [11], the female animals responded with increases in right heart weight and right ventricular pressure only after treatment with SNOAC and hypoxia (Figure 5A, B).

### Serum SNOAC levels are greater in male mice

**Figure 6:**
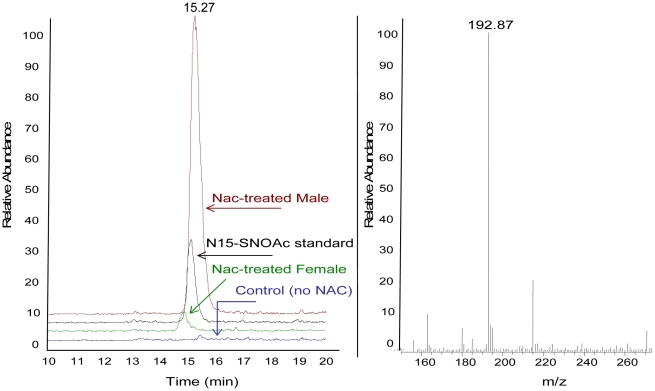
Serum SNOAC levels are lower in female mice. Serum SNOAC was measured by mass spectrometry (MS) in C57BL6/129SvEv male and female mice treated with NAC. Left panel  =  LC chromatogram: Right panel =  MS spectrum. Serum from NAC-treated male (red) and female (green) mice had a SNOAC peak (m/z 193) that co-migrated with the ^15^N-SNOAC standard (black). No signal was seen in non-treated mice (blue). The amount of SNOAC was less in the female mice.

NAC is S-nitrosylated forming SNOAC in the plasma [11]. To examine the relative levels of SNOAC present in the plasma in male and female animals, blood was harvested from the right ventricle of NAC-treated mice and SNOAC levels determined by mass spectroscopy. The level of SNOAC found in the plasma of NAC treated female animals was significantly less than SNOAC levels found in the plasma obtained from NAC-treated male animals (Figure 6). None the less, exogenous administration of SNOAC still resulted in increases in right ventricular pressure and right ventricular weight characteristic of PH (Figure 5) In contrast, analysis of the total amount of endogenously produced S-nitrosothiols in whole blood taken from the left ventricle of untreated male and female mice demonstrate no significant differences in the levels of S-nitrosothiols (Table 4).

**Table 4:** SNO/Hb levels are similar in untreated Male and Female C57Bl6/129SvEv Mice.

Gender	SNO/Hb
Male	7.75×10^−5^±2.49×10^−5^
Female	8.75×10^−5^±1.50×10^−5^

Left ventricular blood was collected from male (n = 4) and female (n = 4) mice and subjected to reductive chemiluminescence in the presence of carbon monoxide (3C assay). SNO values were normalized to hemoglobin content.

### Castration increases GSNO-R activity and protects males from NAC-induced pathology

**Figure 7:**
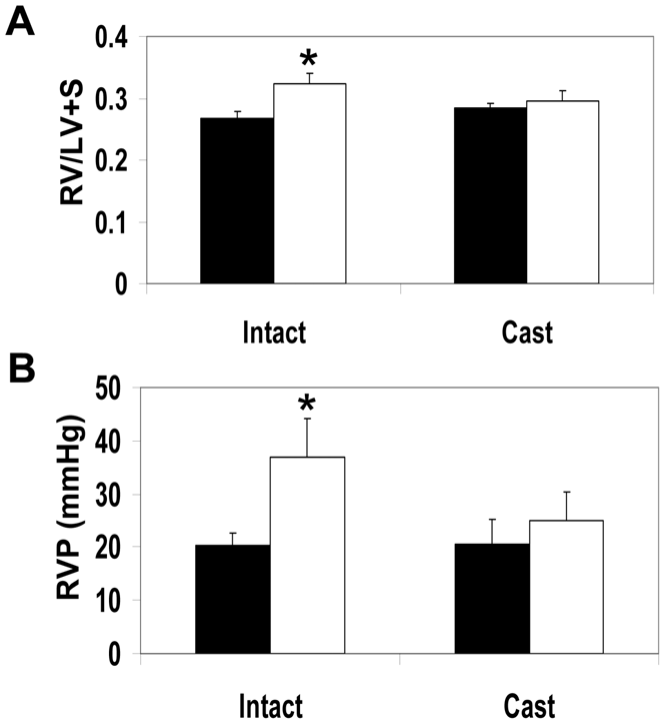
Castration increases GSNO-R activity, protecting against NAC-induced increases in right heart weight and ventricular pressure. Male C57BL6/129SvEv mice with either intact gonads or castrated were treated with (white bars) or without NAC (black bars) for 3 weeks. (A) RVW and (B) RVP were measured. Castration eliminated the increase in both RVW and RVP (N = 11 intact, N = 10 castrated, p<0.001).

**Figure 8:**
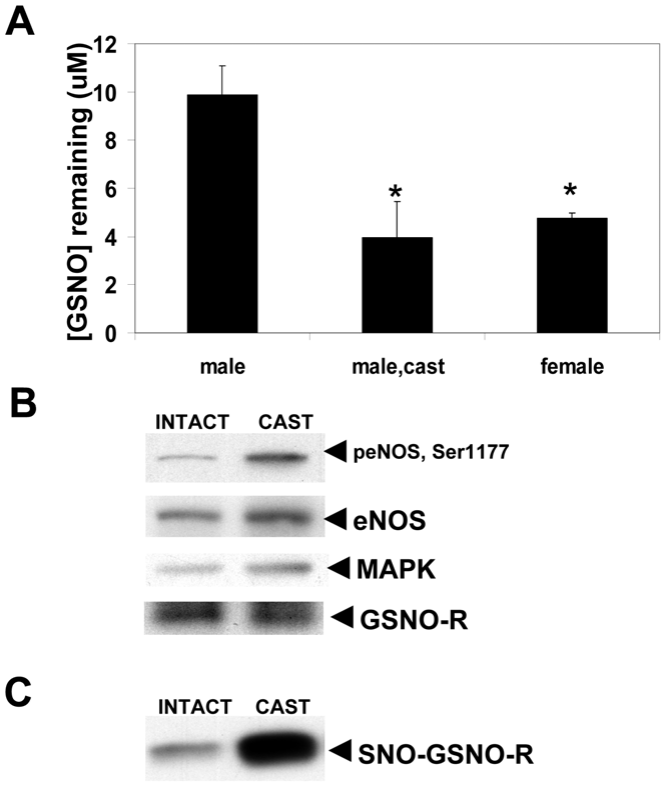
Castration increases GSNO-R S-nitrosylation and activity. (A) Castrated mice show an increase in GSNO-R activity compared to the male gonad intact animals. It should be noted that the levels of GSNO-R activity seen in the castrated mice is similar to that seen in the female mice. The data are presented as the amount of GSNO remaining after 5 min as determined by LC/MS. (B) GSNO-R protein expression in gonad intact and castrated animals was determined by western blot. No differences in GSNO-R protein expression were seen in the intact or castrated animals. (C) S-nitrosylated GSNO-R (SNO-GSNO-R) was determined by biotin switch followed by western blot analysis using anti-GSNO-R antibodies. S-nitrosylated GSNO-R levels were elevated in the castrated animals.

**Figure 9:**
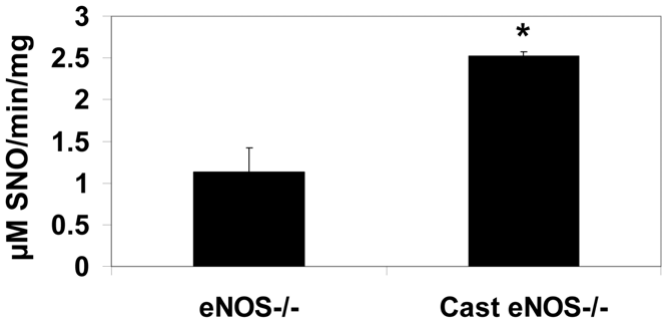
Androgen mediated increases in GSNO-R activity are eNOS-independent. GSNO-R activity was measured in lung homogenates obtained from gonad intact and castrated eNOS^−/−^ mice. Castration resulted in a significant increase in GSNO-R activity as measured by modified Saville Assay using 28 µM GSNO with no protein precipitation. (n = 6 intact, n = 4 castrated, p<0.038).

Male animals develop PH when exposed to chronic systemic administration of NAC [11]. To determine if sex hormones influence the ability of NAC to induce changes in right ventricular weight and right ventricular pressure in mice, the pulmonary effects of NAC were examined in castrated mice (Figure 7A, B). Analysis of right ventricular weight and right ventricular pressure in the castrated animals show no significant difference between in gonad intact and castrated animals under untreated conditions. However, castrated animals were protected from the increases in right ventricular weight and right ventricular pressure in response to NAC. Castration was accompanied by an increase in GSNO-R activity compared to the gonad intact animals as measured by LC/MS (Figure 8A). The increase in GSNO-R activity was not mediated by increases in GSNO-R protein expression (Figure 8B), but to changes in GSNO-R S-nitrosylation (Figure 8C). Moreover, GSNO-R activity in the castrated animals was comparable to that seen in female animals. It should be noted that eNOS phosphorylation at serine 1177 in castrated animals was elevated compared to gonad intact animals (Figure 8B). To further evaluate the role of androgens in the activation of GSNO-R, eNOS^−/−^ animals were castrated and the activity of GSNO-R was evaluated using a modified Saville Assay. Surprisingly, GSNO-R activity in eNOS^−/−^ castrated animals was significantly increased compared to eNOS^−/−^ gonad intact animals (Figure 9) suggesting the presence of an eNOS-independent androgen mediated effect on GSNO-R activity.

## Discussion

Cysteine S-nitrosylation/de-nitrosylation is a regulated process in normal physiology which has parallels to the protein kinase/phosphatase system [1]. All NOS isoforms as well as other proteins have been shown to cause formation of S-nitrosothiol bonds on specific cysteine residues in co-localized proteins and/or cellular peptides. Likewise, specific enzymes and other proteins are responsible for de-nitrosylating these cysteine residues, particularly downstream of transnitrosation reactions between S-nitrosylated proteins and glutathione. One de-nitrosylating enzyme is S-nitrosoglutathione reductase (GSNO-R), a ubiquitously expressed NADH-dependent enzyme. GSNO-R has been shown to be necessary for a variety of effects ranging from the nitrosylation of G-protein coupled receptor kinases to cellular protection from pathological nitrosative stress [1].GSNO-R activity in this manuscript was measured by LC/MS, GSNO-dependent NADH consumption, and modified Saville Assay (Tables 1,2,3). Regardless of the method used to measure GSNO-R activity, GSNO-R activity was found to be approximately 2–3 fold higher in the lung homogenates obtained from female animals. In contrast, lung homogenates obtained from sexually immature mice did not demonstrate any gender specific differences in GSNO-R activity. Moreover, the gender-dependent effects on GSNO-R activity appear to be specific for the lung as GSNO-R activity in kidney and the liver homogenates were not seen. The data, taken together, strongly suggests gender, most likely through the influence gonadal steroids, influences the activity of GSNO-R in the lung.

High-dose, long term systemic treatment with NAC causes PH in male mice by conversion to SNOAC resulting in the upregulation of hypoxia regulated genes [11]. This effect is eNOS-dependent as eNOS^−/−^ mice are completely protected [11]. In this report, we demonstrate: 1) NAC treatment results in lower plasma SNOAC levels in female mice than in male mice, despite increased eNOS expression and 2) female mice exposed to long term NAC treatment do not develop PH in contrast to male mice. We hypothesize that in vivo, females might be protected against eNOS–dependent nitrosative stress through the actions of GSNO-R. This GSNO-R based protection can be overwhelmed by chronic high dose systemic SNOAC treatment or by chronic hypoxia.

Female rats [21], [22] and swine [23] have been shown to develop less severe PAH in response to chronic hypoxia compared to males. Sex hormones may be involved in mediating this gender dependent difference. Estrogen receptors are present in the rat [24] and human lung [25]. Female animals produce more estrogen which is known to enhance the vascular expression and activity of eNOS [22], [25]. This increase in eNOS protein/activity levels is thought to be responsible for the protective effects on the vasculature. In support of this concept, estrogens have been shown to attenuate the severity of PH in rats [22], although the mechanism by which this occurs does not appear to be associated with alterations in eNOS expression [22]. Theoretically, increased eNOS levels produce an increase in S-nitrosylated proteins. Thus, GSNO-R might protect against nitrosative stress, which would be anticipated to be greater in female mice than in males because of the estrogen-mediated increase in eNOS activity. In this report, GSNO-R activity is, indeed, higher in the female mouse lung when compared to the male. Surprisingly, this difference was not driven by a difference in protein expression, but rather by a difference in activity.

Analysis of S-nitrosothiol levels in the serum of untreated normal male and female mice indicate no significant differences in S-nitrosothiol levels, suggesting that the formation and catabolism of S-nitrosothiols is balanced in healthy untreated animals. Because eNOS activity is elevated in the female mouse lung, it is possible that GSNO-R opposes protein S-nitrosylation by eNOS through its de-nitrosylating activity. Thus, the increased eNOS activity present in the female mouse lung might lead to the increased GSNO-R activity as a counter-regulatory mechanism to minimize nitrosative stress. Indeed, in vitro treatment of endothelial cells with GSNO increases GSNO-R activity through S-nitrosylation. This appears to be confirmed in vivo as eNOS^−/−^ mice (reduced S-nitrosothiol formation) have decreased GSNO-R activity which is not due to decreased expression. Moreover, in other maladies, there is evidence for gender discordance in GSNO-R expression and/or activity. For instance, gastric activity of GSNO-R may be a component of the enhanced vulnerability of women to develop alcohol–related diseases [8]. Likewise, gender differences seen in the LPS model of septic shock are eliminated in GSNO-R knockout mice [4]. However, the gender dependent differences in GSNO-R activity in the pulmonary vasculature and the influence this may have on the gender predilection of pulmonary diseases, such as PH have not been examined.

The data suggest that androgens may also have a role in regulating the activity of GSNO-R. Specifically, castration increases GSNO-R activity to a level similar to that seen in female mice. This increase does not appear to be due to increases in protein expression but again reflects increases in GSNO-R S-nitrosylation. Moreover, the resulting increase in GSNO-R activity correlates with protection from NAC-induced PH. The mechanism by which androgens activate GSNO-R is not yet known. The data indicate, on one level, castration increases the expression and activity of eNOS, consistent with the observation that eNOS^−/−^ mice have reduced GSNO-R activity. It also suggests that androgens decrease the expression and activity of eNOS within the pulmonary vasculature. However, the effects of androgens on eNOS expression and activity are in contrast to published data. For instance, in the aorta, androgens have been reported to activate eNOS through a non-genomic pathway mediated by cSrc, PI3K and Akt [26], [27]. Moreover, male androgen receptor knockout mice show decreased aortic eNOS expression and phosphorylated eNOS compared to wild type mice [28]. Lastly, neither androgen receptors nor nitric oxide are involved in the vasodilatory actions in the pulmonary vasculature in response to acute administration of testosterone, a major androgen produced in the testes [29]. The reasons for these discrepancies are unclear, but may be due to the vascular bed studied, or differences mediated by chronic versus acute effects of androgens. On a second level, studies examining the activity of GSNO-R in castrated eNOS^−/−^ mice demonstrate an increase in GSNO-R activity with castration in the absence of eNOS, suggesting that androgens have a separate eNOS-independent mechanism by which they alter GSNO-R activity. Thus, it is possible that the net effect in females in GSNO-R activity is increased, in part, through S-nitrosylation by eNOS as well as by the absence of what appears to be a distinct, androgen-dependent effect.

In the pulmonary vascular bed, evidence has emerged for the following paradigm: increased delivery of nitrosothiols to the pulmonary vascular bed results in the activation of hypoxia related gene regulatory proteins including specificity proteins and hypoxia inducible factor-1 through S-nitrosylation reactions [11]. Downstream, this effect results in the upregulation of hypoxia-regulated genes, leading to cellular and morphologic changes characteristic of PH. Thus, excessive S-nitrosothiol delivery in the pulmonary vasculature recapitulates hypoxia. To some extent, this paradigm can provide insight into PH of diverse causes: 1) hypoxia results in transnitrosation from deoxyhemoglobin to glutathione and other thiols in the systemic periphery which then return in erythrocytes and plasma and are dumped into the pulmonary vascular bed causing PH by activating hypoxia responsive genes; 2) to the extent that normal levels of deoxygenation increase systemic vascular S-nitrosothiols, a tripling of venous return in high flow states triples the burden of S-nitrosothiols returning to the lung, perhaps explaining high flow state-induced PH; 3) systemic inflammation can increase erythrocytic S-nitrosothiol load, contributing to the risk of PH seen in systemic inflammation. Note the paradox: S-nitrosothiols delivered acutely to the pulmonary vascular bed are vasodilators and will reverse PH; whereas, chronic exposure of the pulmonary vascular bed to S-nitrosothiols gradually causes pulmonary vascular remodeling and chronic pulmonary hypertension. If increased GSNO-R activity in female mice can protect against the adverse affects of nitrosative stress associated with eNOS activation, chronic inflammation, chronic hypoxemia and high flow states, why are female humans at higher risk for the development of PH from a variety of causes than are males? The answer may lie in GSNO-R itself. In the absence of androgens and in the presence of eNOS activation, increased GSNO-R activity is protective. However, this system leaves females particularly vulnerable to GSNO-R dysfunction. For example, if there is decreased GSNO-R activation by eNOS or an androgen-mimetic effect to decrease GSNO-R activity in the female pulmonary vascular endothelium, by virtue of increased eNOS activity and the resulting chronic nitrosative stress, females will be at increased risk for hypoxia-mimetic pulmonary vascular remodeling. Indeed, there are single nucleotide polymorphisms resulting in increased or decreased GSNO-R activity that appear to be associated with both increased and decreased asthma risk respectively [30]. These data suggest that future studies regarding GSNO-R expression, genetics and activity in the lungs of women with PH may be worthwhile.

In summary, GSNO-R activity is increased post-translationally in the female murine lung. This observation is important because it suggests that GSNO-R can protect against chronic nitrosative stress associated with increased eNOS expression in female mice. This may be particularly important in protecting against the high flow state and chronic eNOS upregulation associated with mammalian pregnancy [31], [32]. In addition, it may also help to explain why females, in general, are more tolerant of high altitude living than males [33], [34]. However, an extension of these murine results to human studies has not yet been undertaken.

## Materials and Methods

### Materials

All chemical reagents used in the studies were obtained from Sigma Chemical Company (St. Louis, MO) unless otherwise stated.

### Animals

All procedures in the treatment and care of the animals used in this study were approved by the Animal Care and Use Committee at the University of Virginia. Female C57BL6/129SvEv mice (10-12 w) were exposed to normoxia, 52 mM N-acetyl cysteine, 1 mM S-nitrosylated N-acetyl cysteine, or 10% oxygen for 3 weeks as previously described [11]. Castrations were performed when animals reached 3 w of age. All animals were anesthetized using interperotineal injection of ketamine/xylazine (60–80/5–10 mg/kg) prior to the procedure. In addition, the surgical site was made aseptic using betadine solution and 70% ethyl alcohol. Local anesthesia at the incision site was by infiltration of 0.1 ml 0.25% bipivicaine. For castration, a small incision was made at the tip of the scrotum. The tunic opened and the testis, cauda epidiymis, vas deferens and the spermatic blood vessels exteriorized. The blood vessels and the vas deferens were cauterized and the testis and epididymis removed. The remaining tissue was returned into the sac and the procedure repeated for the other testis. The skin incision was closed with Nexaband. Mice are kept warm after surgery until they are responsive to stimuli and monitored until they can completely right themselves on their own. Right ventricular pressure was measured in anesthetized mice using a 1.4 F Millar catheter/transducer as previously described [11]. Right heart hypertrophy was measured as a ratio of the weight of the right ventricle/weight of the left ventricle + septum.

### Measurement of S-nitrosoglutathione reductase activity

LC/MS: Lung homogenate (0.25–0.5 µg) in homogenation buffer was incubated with GSNO (28 µM) and 2 mM GSH in the presence or absence of 300 µM NADH at 37°C for 5 minutes. Protein was precipitated from the reaction using 8% tricholoracetic acid. HPLC analysis was performed on a Waters 2695 separation module using a Waters Symmetry C8 (2.1×150 mm) column. GSNO analytes were eluted using an isocratic method of 95% formic acid (0.1%) in water and 5% methanol. Mass spectroscopy was performed using a Finnigan LCQ ion trap mass spectrometer equipped with an electrospray ionization source. Data were collected in positive ion mode with selective ion monitoring at m/z = 336.5–337.5 and quantified against GS^15^NO cations m/z = 337.5–338.5. Integrated peak areas were determined using Xcalibur software. GSNO-dependent NADH Consumption [35]: Cell lysate (0.3 ug/ml) was incubated with 75 µM NADH in reaction buffer (20 mM Tris HCL pH 8.0 and 0.5 mM EDTA containing 0 or 100 µM GSNO at room temperature. NADH fluorescence (absorption at 340 nm and emission at 455 mm) was measured over time in a FLUOstar Omega (BMG Labtech, Offenburg, Germany). Concentration of NADH was determined from a standard curve. Modified Saville Assay: GSNO-R activity was measured by modified Saville Assay. Briefly, 250 µg of cell lysate or lung homogenate was incubated in the absence or presence of 300 µM NADH, in the presence of 2 mM GSH and 28 µM or 100 µM GSNO. Assay was performed with and without protein precipitation (Table 3). Two aliquots of 75 µl were placed into a 96 well plate at 1 min intervals for a total of 5 min. One aliquot was placed with 75 µl of (+) reagent (58 mM Sulfanilamide +7.36 mM HgCl_2_ in 1 N HCl) while the second aliquot was placed with (−) reagent (58 mM Sulfanilamide in 1 N HCl). Samples were incubated 5 min in the dark. At the end of this incubation, 75 µl of (N) reagent (0.77 M n-(1-napthyl) ethylene-diamine dihydrochloride) was added. Samples were incubated 5–10 min for color to develop. Absorbance was read at 540 nm. Amount of GSNO remaining in the reaction was determined from a GSNO standard curve. Activity was obtained from the slope of the time course divided by the amount of protein in the reaction.

### Measurement of S-nitroso-N-acetyl cysteine (SNOAC)

The abundance of SNOAC was determined by liquid chromatography/mass spectroscopy as previously described [11].

### Immunoprecipitation

Cell lysate or lung homogenate (100–300 µg) prepared in lysis buffer (20 mM Tris pH 7.6, 150 mM NaCl, 2 mM EDTA, 10%glycerol 1%Triton X-100 +1X proteinase inhibiters) were immunoprecipitated using 4 µg anti-eNOS antibody (BD Biosciences, San Diego) overnight at 4°C on a rotator. Samples were incubated with 100 µl protein A agarose beads (Roche Diagnostics, Mannheim, Germany) for 2–4 hours at 4°C. At the end of the incubation, the agarose beads were spun at 3000×g for 1 min and washed three times with lysis buffer. Protein was eluted from the protein A agarose beads by incubation with 50 µl SDS loading buffer.

### Biotin-Switch

The biotin switch procedure for the identification of S-nitrosothiols was performed to identify S-nitrosylated proteins [36]. Briefly, cell lysate or lung homogenate (100–300 µg) were precipitated using acetone and resuspended in 100 µl HEN Buffer (250 mM HEPES pH 7.7, 1 mM EDTA, 0.1 mM neocuproine). Samples were mixed with 4 volumes Blocking Buffer (9 vol HEN+1 vol 25%SDS containing 20 mM MMTS) and incubated at 50°C for 20 min with shaking. Protein was precipitated by acetone, resuspended in HEN Buffer containing 1 mM Biotin-HPDP (Pierce) and 1 mM ascorbate and incubated for 1 h at room temperature. Biotin HPDP was removed by precipitation with acetone and pellet resuspended in 100 µl HEN buffer. Resuspended sample was neutralized using Neutralization Buffer (20 mM HEPES pH 7.7, 10 mm NaCl, 1 mM EDTA, 0.5% Triton X-100.) Biotinylated proteins were isolated by incubation with Streptavidin agarose for 1 h at room temperature. Resin was washed 5 times with Neutralization Buffer containing 600 mM NaCl and biotinylated protein eluted with 50 µl 2X SDS PAGE Loading Buffer.

### Western Blot analysis

Proteins were separated on a 10% polyacrylamide gel as previously described [11]. Proteins were transferred to Immobilon-P transfer membranes (Billerica, MA) and signal detected using SuperSignal West Pico Chemiluminescent Substrate (Thermo Scientific, Rockford, IL).

### Statistics

All data will be expressed as the mean ± SEM. All statistical analysis was performed using Sigma Stat software. Data comparing 2 groups were analyzed using a paired T test, with p<0.05 considered significant. Data comparing more than one group will be analyzed by one way ANOVA. Significance was determined using the Holm Sidak post hoc test with p<0.05 considered significant.
